# Comparing Physician Assistant and Nurse Practitioner Practice in U.S. Emergency Departments, 2010–2017

**DOI:** 10.5811/westjem.2021.5.51988

**Published:** 2021-08-21

**Authors:** Fred Wu, Michael A. Darracq

**Affiliations:** University of California, San Francisco, Department of Emergency Medicine, Fresno, California

## Abstract

**Introduction:**

We sought to compare physician assistant (PA) and nurse practitioner (NP) practice in United States emergency departments (ED) based on ED visits as reported by the National Hospital Ambulatory Medical Care Survey (NHAMCS).

**Methods:**

We performed a retrospective, secondary analysis of the 2010 to 2017 NHAMCS with analysis of ED visits, patient demographics, and hospital characteristics.

**Results:**

Between 2010 to 2017, 21.0% (95% confidence interval, [CI] +/−3.1%) of ED visits were seen by either a PA/NP (with and without physician involvement) and 8.6% (+/−2.9%) were seen by PA/NP alone. We identified an increase for NP visits between 2014–2016 and found that PA/NP visits share many of the same characteristics.

**Conclusion:**

While emergency medicine has predominately been a specialty for PAs, the number of ED visits with NPs has been increasing over the past several years. While there are some differences, PAs/NPs share many similar practice characteristics in the ED.

## INTRODUCTION

Physician assistants (PA) and nurse practitioners (NP), commonly referred to as advanced practice clinicians, advanced practice providers or midlevel providers, are increasingly being used in US emergency departments (ED) and as a result are causing some controversy. Some have expressed concern that PAs and NPs are replacing emergency physicians with associated financial repercussions. Published literature regarding PA and NP “replacement” is generally anecdotal, without objective data, or applicable analysis.^1^–^3^

Approximately 14,000 NPs (representing 5.9% of the total US-licensed NPs) practice in the acute care setting according to the American Association of Nurse Practioners.^4^ Approximately 13% of certified PAs (which represents over 12,000 PAs) practice emergency medicine (EM).^5^ In 2009 an estimated 77.2% of US EDs used PAs and NPs in day-to-day patient care.^6^ According to the Emergency Department Benchmarking Alliance, a 39% increase in the use of NPs and PAs was observed between 2010–2016 among US EDs.^7^ A secondary analysis of 2014 Medicare data determined that the ED workforce consisted of 58,641 clinicians with 24.5% classified as advanced practice providers; 68.4% of these were PAs, and 31.5% were NPs.^8^

PAs and NPs have different clinical practice pathways.^9^ PAs are educated along a medical model similar to US medical students, while NPs are educated along a nursing model.^10^ PAs and NPs also have different scopes of practice, practice theories, and educational models.^10^,^11^ Independent practice as described by Full Practice Authority eliminates unnecessary contracts or agreements with physicians, along with elimination of oversight by the state medical board, and is supported by the American Association of Nurse Practitioners.^12^ In 2019, 28 states and the District of Columbia granted NPs Full Practice Authority to practice without physician supervision.^13^ The American Academy of Physician Assistants also supports the elimination of a legal requirement for a specific relationship between a PA and a physician.^14^

Prior studies analyzing the use of advanced practice or midlevel providers in the ED have not distinguished between NPs and PAs but rather present data in aggregate as “midlevel providers.”^6^,^15^,^16^ These previous studies have not directly compared PA to NP utilization in US EDs. Thus, we believe comparison of these practice pathways in the ED is appropriate given the differences in education; desired scope of practice; practice theories; the absence of previous comparisons of PA and NP utilization in the published literature using National Hospital Ambulatory Medical Care Survey (NHAMCS) data; and the controversy regarding utilization of midlevel providers. We sought to compare PA to NP utilization in US EDs from 2010–2017 using publicly available data from the NHAMCS.^17^ Specifically, we sought to compare ED visits with physician involvement (PA with physician, NP with physician) and without physician involvement (PA only, NP only). We analyzed patient demographics and visit and hospital characteristics.

## METHODS

The study methodology, including data analysis, is similar to that in a previously published paper in which we used NHAMCS data to compare PA ED visits with and without physician involvement to physician-only visits.^18^

### Study Design

The institutional review board reviewed and approved this study within an exempt protocol. The NHAMCS collects data on the utilization and delivery of ambulatory care services in hospital EDs. This initiative is sponsored by the US Centers for Disease Control and Prevention, National Center for Health Statistics. Each year since 1992 a four-stage probability sample of representative hospitals, exclusive of federal, military, and Veteran’s Administration hospitals, located in the 50 states and District of Columbia are identified to provide data on a sample of ED patient visits over a four-week reporting period. During this reporting period, onsite interviewers collect data on a computerized patient record form. The collected data include patient characteristics such as age, gender, race, ethnicity, along with visit characteristics such as patient’s reason for visit, provider’s diagnosis, service ordered or provided, and treatment including medications. Facility data are also collected. Because it is a representative sample, the collected data are weighted to produce national estimates. Further methodological details for the NHAMCS have been published elsewhere.^19^ The NHAMCS is also endorsed by multiple EM organizations.^20^ This current study is a retrospective secondary analysis based on a validated, national, cross-sectional survey.

### Study Protocol

The NHAMCS data (available at https://www.cdc.gov/nchs/ahcd/datasets_documentation_related.htm) was downloaded and converted using SPSS Statistics version 25 (IBM Corporation, Armonk, NY). We queried the NHAMCS survey variable “provider seen” to identify all patient visits seen by PA or NP with or without physician involvement. Although the dataset is extensive with multiple data points, we focused on demographic data including age, gender, race, ethnicity, insurance status, mode of arrival, acuity, diagnostic studies ordered (imaging and/or laboratory studies), procedures performed, ED length of stay, ED disposition, and hospital geographic region. The acuity was determined by a triage nurse when the patient presented to the ED, with patients assigned a number from 1–5 (1=immediate, 2=emergent, 3=urgent, 4=semi-urgent, 5=non-urgent). Emergency departments with a 3- or 4-level acuity system were rescaled to fit the 5-level system. The PA data presented here is the same as in a previously published manuscript.^18^

**Population Health Research Capsule**

What do we already know about this issue?

*Physician assistants (PAs) and nurse practitioners (NPs) are widely used in US emergency departments (EDs). There has been no published work comparing the two groups using a national database*.

What was the research question?

*To compare PA and NP utilization (with and without physician involvement) in US EDs from 2010 to 2017 using a national database*.

What was the major finding of the study?

*NP utilization has significantly increased over this time. Practice characteristics are similar between the two groups. Between 2010 to 2017, 21.0% (95% confidence interval, [CI] +/*−*3.1%) of ED visits were seen by either a PA/NP (with and without physician involvement) and 8.6% (+/*−*2.9%) were seen by PA/NP alone*.

How does this improve population health?

*There is concern that PAs/NPs are caring for patients independently. Nearly 60% of PA/NP ED visits are co-managed with physicians*.

### Data Analysis

We calculated descriptive statistics, including sample standard errors, using IBM SPSS Statistics Complex Samples module. As described in the 2015 NHAMCS micro-data file documentation Appendix 1 (https://data.nber.org/nhamcs/docs/nhamcsed2015.pdf), the stratum variable, the cluster variable, and the weighting variable were used to calculate the descriptive statistics. We used the standard errors to calculate 95% confidence intervals (CI), which are presented to aid in the interpretation of the results.

## RESULTS

An estimated one billion ED visits took place between 2010–2017. Five percent (CI, 2.3–7.7] of these visits were seen by a PA only; 8.2% (CI, 5.5–10.9) by a PA with physician involvement; 3.6% (CI, 0.7–6.5) by a NP only; and 4.2% (CI, 1.1–7.3) by a NP with physician involvement. There was a 7% increase in ED volume between 2010–2017. There was no difference in PA-only visits compared to NP-only visits (5.0% [CI, 2.3–7.7] v 3.6% [CI, 0.7–6.5]). There was a difference in PA with physician involvement visits compared to NP with physician involvement visits (8.2% [CI, 5.5–10.9] v 4.2% (CI, 1.1–7.3); *P* <0.001].

**Figure 1:**
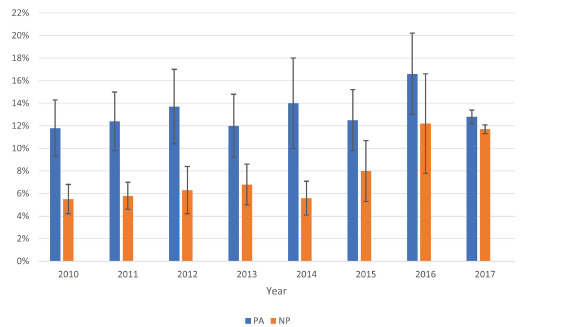
Percentage of US emergency department visits seen by physician assistants (PA) or nurse practitioners (NP), 2010–2017. Error bars represent 95% confidence intervals for annual estimates; PA or NP visits include with and without physician involvement.

Figure 1 shows the percentage of US ED visits seen by either a PA or a NP, which includes visits with and without physician involvement, between 2010–2017. There was no difference in percentage of visits for PA visits when comparing 2010 to 2017 (11.8% [CI, 9.3–14.3] v 12.8% [CI, 12.1–13.4]). There was a difference of 6.2% in percentage of visits for NP visits when comparing 2010 to 2017 (5.5% [CI, 4.2–6.8] v 11.7% [CI, 11.3–12.1]; *P* <0.001).

Table 1 displays aggregate patient and visit characteristics of ED visits by provider seen. Approximately 33% of the patients cared for by PA-only visits or PA with physician involvement were patients 25–44 years old. We observed no difference between patients 0–15 years old and 25–44 years old (28.8% [CI, 21.0–36.7] v 28.5% [CI, 20.6–36.4]) for patients cared for by a NP only. Individuals 25–44 years old (28.9%) comprised the most common cohort among patients cared for by NPs with physician involvement. More than 90% of NP-only and PA-only visits were for patients less than 65 years of age. More than 80% of PA with physician and NP with physician visits were for patients less than 65 years of age. Approximately 50% of visits by PAs and NPs were for patients with public insurance. Between 2010–2017, we observed no difference in the percentage of ambulance arrivals being cared for by PA only compared to NP only (5.8% [CI, 4.5–7.1] v 4.9% [CI, 3.1–6.7]). Similarly, no difference was observed between ambulance arrivals for PA with physician compared to NP with physician (14.7% [CI, 12.1–17.4] v 13.1% [CI, 10.4–15.8]).

The most common acuity seen by PA-only and NP-only visits was for semi-urgent/non-urgent patients (56.4% [CI, 45.7–67.1]) and 48.8% [CI, 39.2–58.4]). A difference in immediate/emergent acuity, the sickest patients, was observed between PA only and NP only (3.2% [CI, 2.2–4.2] v 2.1% [CI, 1.2–3.0]; *P* <0.001]. There was also a difference in the percentage of urgent acuity seen by PA only compared to NP only (24.7% [CI, 19.1–30.] v 18.0% [CI, 13.0–23.0]; *P* <0.001]. No difference was observed between frequency of diagnostic screening, imaging, procedures performed, and medications ordered between PA-only and NP-only visits. This same pattern was also found when physicians were involved with PA and NP care. Hospital admission rates were similar between PAs and NPs. Most PA-only and NP-only visits resulted in a length of stay between 1–1.9 hours (32.9% [CI, 26.2–39.6} and 34.3% [CI, 26.7–41.9], respectively). More than one third of PA and NP ED visits occurred in the Southern US.

## DISCUSSION

We sought to compare PA vs NP utilization between 2010–2017 using NHAMCS data to analyze trends in patients seen by provider type, patient demographics, visit characteristics, and hospital characteristics. Between 2010–2017, the number of ED visits involving NPs increased by greater than twofold (a 6.2% increase overall). As ED volume increased by 7.0% within this time, the increase in ED visits involving NPs nearly matches it. By 2017 there was a small difference between ED visits involving PAs vs NPs, which may indicate a narrowing of the gap. Between 2010–2017, there were more visits involving PAs alone than visits involving NPs alone. This same period shows an increase in PA with physician visits compared to NP with physician visits. The majority of ED visits involving PAs or NPs were for semi-urgent/non-urgent visits. PA and NP visits share many of the same characteristics such as diagnostic screening, imaging ordered, procedures performed, admission rate, and ED length of stay.

The cause of the increase in NP visits between 2010–2017 is not known. Further study is required to determine whether factors such as NP Full Practice Authority, hiring by administrators instead of physicians, or other reasons are responsible for the current trend. The NP supply may also be a significant contributing factor as more than 30,000 new NPs graduated in 2018–2019 compared to over 9000 PA graduates in 2018.^21^,^22^ However, determining why NP visits increased between 2010–2017 was not the primary purpose of the study, and the above factors are not contained within the NHAMCS data.

Another concern among some is the perception that PAs and NPs are increasingly caring for higher acuity patients.^2^,^3^,^23^ According to the results of the present survey, the majority of ED visits involving PAs or NPs are for semi-urgent/non-urgent visits, while caring for immediate/emergent visits represents the minority of ED visits. When PAs or NPs are involved with immediate/emergent visits, a statistically significant number of those visits involve PA or NP with physician rather than visits by PA or NP alone. Also, PAs and NPs mostly cared for patients younger than 65 years old. Patients older than 65 traditionally have more co-morbidities and may be more complex or with higher acuity.

In 2018 Phillips et al examined PA and NP practice patterns in the ED.^24^ They reported on the results of a survey administered to the American College of Emergency Physicians’ council, which showed that NPs used more resources than PAs, regardless of years of experience. Our review of the NHAMCS data shows no difference between PAs and NPs, with and without physician involvement, regarding diagnostic screening, imaging ordered, procedures performed, and medications ordered. Besides reporting that NPs use more resources than PAs, Phillips et al also report from their physician survey that NPs needed additional clinical training more often than PAs and that EDs are more willing to hire less-experienced PAs than less-experienced NPs; thus concluding that PAs have more favorable work characteristics. Given this perspective by a group of EM leaders, it is interesting to note the growth of NPs within EM, a specialty traditionally staffed by a PA majority. In 2017, NP utilization nearly caught up to PA utilization and the difference was only by a small margin.

## LIMITATIONS

The NHAMCS dataset is widely used by researchers to report various ED clinical conditions and characteristics. Unfortunately, as a survey, there are limitations such as errors in data collection and coding, which may alter interpretations and final conclusions. As described earlier, NHAMCS used to use paper instruments, where poor handwriting may have limited interpretation; however, those issues should have been resolved when computer versions of the survey were introduced after 2012. Coding and data errors are limited with trained research and survey staff but not completely eliminated. Surveyors may also not know with certainty which provider group was directly or indirectly involved with the patient’s care and whether “provider seen” is discussion or actual physical examination of the patient. However, this appears to be a consistent limitation throughout the surveys.

We were not involved in ED survey site selection, but it is generally accepted that these sites are representative of US EDs. Neither were we involved in determining the weighting process used to produce national estimates. These limitations have been described in a previous study using the NHAMCS dataset.^18^ Cooper also expressed these concerns and others when using the NHAMCS dataset.^25^

## CONCLUSION

From 2010 to 2017, physician assistants and nurse practitionerss were involved with 21% of US ED visits. While EM has predominately been a specialty for PAs, the number of NPs has been increasing over the past several years. In fact, there has been a greater than twofold increase in the number of visits seen by NPs between 2010–2017. PA and NP visits share many of the same characteristics such as patient age, gender, insurance status, arrival by ambulance, diagnostic screening, procedures performed, imaging ordered, admission rate, and ED length of stay. Further study will be needed to determine whether these trends continue.

## Figures and Tables

**Table 1:** Characteristics of emergency department visits seen by physician assistant (PA) only, PA with physician, nurse practitioner (NP) only and NP with physician; 2010–2017.

Characteristics	PA Only	PA with Physician	NP Only	NP with Physician
Total ED visits	5.0 (2.3–7.7)	8.2 (5.5–10.9)	3.6 (0.7–6.5)	4.2 (1.1–7.3)
Patient characterisitcs				
Age (year)				
0–15	20.4 (16.0–24.9)	14.9 (12.0–17.9)	28.8 (21.0–36.7)	18.8 (14.7–22.9)
15–24	19.2 (15.5–22.9)	15.6 (12.8–18.4)	18.5 (12.7–24.2)	15.5 (12.4–18.5)
25–44	32.9 (26.7–39.1)	30.4 (25.5–35.3)	28.5 (20.6–36.4)	28.9 (23.3–34.5)
45–64	19.0 (15.5–22.5)	24.5 (20.7–28.3)	16.7 (12.1–21.3)	21.9 (17.8–26.1)
65–74	4.8 (3.7–6.0)	6.7 (5.4–7.9)	3.7 (1.8–5.7)	6.4 (4.8–7.9)
≥75	3.6 (2.8–4.5)	7.9 (6.4–9.4)	3.8 (1.4–6.2)	8.6 (6.6–10.6)
Female gender	54.1 (44.4–63.7)	55.6 (46.4–64.8)	54.1 (39.5–68.7)	56.4 (46.1–66.6)
Race/ethnicity				
Non-Hispanic White	57.9 (47.4–68.3)	58.2 (46.6–69.8)	55.8 (38.5–73.1)	60.2 (48.4–72.0)
Non-Hispanic Black	23.3 (16.9–29.7)	22.4 (18.3–26.5)	24.7 (12.9–36.4)	23.9 (17.4–30.4)
Hispanic	13.9 (11.0–16.9)	12.8 (10.5–15.1)	12.6 (9.7–15.4)	14.9 (11.8–18.1)
Insurance				
Private	27.2 (21.6–32.7)	29.2 (23.6–34.8)	22.8 (16.9–28.8)	26.5 (22.0–31.0)
Public	46.2 (37.9–54.5)	46.7 (41.7–51.7)	46.9 (37.5–56.3)	48.7 (41.2–56.2)
Self-pay	13.1 (10.5–15.8)	10.8 (8.6–12.9)	13.4 (8.8–17.9)	10.7 (8.4–13.1)
Other/unknown	12.6 (9.2–16)	12.5 (10.2–14.8)	15.7 (5.0–26.4)	12.5 (9.2–15.8)
Visit characteristics				
Arrival by ambulance urgency	5.8 (4.5–7.1)	14.7 (12.1–17.4)	4.9 (3.1–6.7)	13.1 (10.4–15.8)
Immediate/emergent	3.2 (2.2–4.2)	9.1 (7.4–10.8)	2.1 (1.2–3.0)	8.0 (6.3–9.7)
Urgent	24.7 (19.1–30.3)	36.1 (29.4–42.8)	18.0 (13.0–23.0)	34.2 (28.0–40.5)
Semi-urgent/non-urgent	56.4 (45.7–67.1)	37.5 (31.5–43.5)	48.8 (39.2–58.4)	34.6 (29.0–40.2)
No triage/unknown	14.5 (9.0–20.0)	17.3 (13.4–21.2)	29.6 (8.4–50.8)	21.1 (14.8–27.4)
Diagnostic Screening	53.3 (43.1–63.5)	65.1 (54.7–75.6)	54.1 (38.2–69.9)	62.6 (51.9–73.2)
Any imaging	38.8 (32.2–45.4)	53.2 (45.1–61.4)	36.8 (24.8–48.8)	55.6 (45.4–65.7)
Any procedures performed	39.8 (32.8–46.8)	50.0 (42.2–57.9)	38.7 (26.3–51.1)	47.6 (39.0–56.3)
Any medications ordered	71.1 (57.9–84.2)	72.9 (61.3–84.4)	69.4 (52.3–86.4)	68.1 (56.8–79.4)
ED LOS (hours)				
<1	20.4 (15.7–25.2)	14.8 (10.4–19.3)	22.1 (16.4–27.8)	12.5 (10.0–15.0)
1 – 1.9	32.9 (26.2–39.6)	22.4 (8.5–26.3)	34.3 (26.7–41.9)	21.9 (17.9–26.0)
2 – 2.9	21.3 (17.5–25.0)	19.3 (15.9–22.8)	21.3 (15.8–26.9)	20.5 (15.9–25.1)
≥3	25.4 (20.3–30.5)	43.5 (35.9–51.0)	22.2 (17.3–27.2)	45.1 (36.1–54.1)
Hospital admission	1.7 (1.1–2.4)	11.1 (9.0–13.1)	1.5 (0.7–2.4)	11.1 (8.5–13.8)
Hospital characteristics				
US region				
Northeast	18.7 (14.1–23.4)	25.8 (19.4–32.2)	11.7 (8.1–15.4)	16.7 (11.2–22.2)
Midwest	26.3 (17.3–35.3)	28.0 (17.9–38.1)	36.6 (16.6–56.6)	18.3 (13.0–23.6)
South	34.6 (24.5–44.8)	34.0 (24.3–43.6)	38.0 (20.7–55.3)	41.1 (29.8–52.4)
West	20.3 (10.3–30.4)	12.3 (8.2–16.3)	13.7 (7.1–20.2)	23.9 (12.6–35.3)

Data reported as % (95% CI).

*ED*, emergency department; *LOS*, length of stay; *CI*, confidence interval.

## References

[1] The White Coat Investor (2018). Midlevel providers taking physician jobs – Financial impact.

[2] The Student Doctor Network (2019). AAEM Position on APPs.

[3] Emergency Medicine News (2019). After the match: Invasion of the physician assistants.

[4] American Association of Nurse Practitioners (2018). Number of nurse practitioners hits new record high.

[5] National Commission on Certification of Physician Assistants, Inc. (2018). 2018 Statistical profile of certified physician assistants by specialty: An annual report of the National Commission on Certification of Physician Assistants.

[6] Menchine MD, Wiechmann W, Rudkin S (2009). Trends in midlevel provider utilization in emergency departments from 1997 to 2006. Acad Emerg Med.

[7] ACEP Now, American College of Emergency Physicians (2017). More advanced practice providers working in emergency departments.

[8] Hall MK, Burns K, Carius M (2018). State of the national emergency department workforce: Who provides care where?. Ann Emerg Med.

[9] Hass V (2016). Physician assistants and nurse practitioners are not interchangeable. JAAPA.

[10] Bednar S, Atwater A, Keough V (2007). Educational preparation of nurse practitioners and physician assistants: an exploratory review. Adv Emerg Nurs J.

[11] EP Monthly (2014). Mid-level providers: Who they are, what they do, and why they’re changing emergency medicine.

[12] American Association of Nurse Practitioners (2019). Issues at a Glance: Full Practice Authority.

[13] California Health Care Foundation (2019). Expanding the role of nurse practitioners in California: physician oversight in other states.

[14] American Academy of PAs Optimal team practice. https://www.aapa.org/advocacy-central/optimal-team-practice/.

[15] Wiler JL, Rooks SP, Ginde AA (2012). Update on midlevel provider utilization in U.S. emergency departments, 2006 to 2009. Acad Emerg Med.

[16] Ginde AA, Espinola JA, Sullivan AF (2010). Use of midlevel providers in US EDs, 1993 to 2005: implications for the workforce. Am J Emerg Med.

[17] National Center for Health Statistics, Centers for Disease Control and Prevention (2019). About the ambulatory health care surveys.

[18] Wu F, Darracq MA (2020). Physician assistant utilization in U.S. emergency departments; 2010 to 2017. Am J Emerg Med.

[19] National Center for Health Statistics, Centers for Disease Control and Prevention (2019). Survey methods and analytic guidelines.

[20] National Center for Health Statistics, Centers for Disease Control and Prevention (2020). Ambulatory health care data – Professional endorsements.

[21] American Association of Colleges of Nursing (AACN) (2020). 2019–2020 Enrollment and Graduations in Baccalaureate and Graduate Programs in Nursing.

[22] Physician Assistant Education Association (2018). By the numbers: Program report 34: Data from the 2018 program survey.

[23] Emergency Medicine News (2017). Are NPs and PAs taking EP jobs?.

[24] Phillips AW, Klauer KM, Kessler CS (2018). Emergency physician evaluation of PA and NP practice patterns. JAAPA.

[25] Cooper RJ (2012). NHAMCS: does it hold up to scrutiny?. Ann Emerg Med.

